# Research progress on immunotherapy combined with neoadjuvant concurrent chemoradiotherapy in pMMR/MSS locally advanced rectal cancer

**DOI:** 10.3389/fimmu.2025.1631620

**Published:** 2025-08-08

**Authors:** Yang He, Wendong Gu, Yingjie Shao

**Affiliations:** Department of Radiation Oncology, The Third Affiliated Hospital of Soochow University, Changzhou, China

**Keywords:** combination therapy, immune checkpoint inhibitors, locally advanced rectal cancer, neoadjuvant chemoradiotherapy, MSS

## Abstract

Locally advanced rectal cancer (LARC) constitutes a particularly challenging subtype of rectal cancer. Although traditional neoadjuvant chemoradiotherapy (nCRT) has demonstrated efficacy in enhancing local disease control and promoting sphincter preservation, its impact on long-term survival outcomes remains suboptimal. In recent years, combinatorial approaches integrating immune checkpoint inhibitors (ICIs) with nCRT have garnered increasing research interest. Nevertheless, individuals with proficient mismatch repair (pMMR)/microsatellite stable (MSS) LARC exhibit a notable resistance to immunotherapeutic strategies. This review thoroughly assesses the molecular features and treatment challenges linked to pMMR/MSS LARC, elucidates the functional pathways of ICIs, and explores their prospective synergistic effects when administered alongside nCRT. Moreover, recent progress in clinical investigations is summarized, and the utility of emerging biomarkers in facilitating patient selection and assessing treatment efficacy is critically appraised.

## 1 Introduction

Global cancer statistics demonstrate that rectal cancer (RC) sits among the malignancies with relatively high incidence and mortality rates worldwide (1). Although early detection rates have improved due to ongoing advancements in screening and diagnostic technologies, managing locally advanced rectal cancer (LARC) continues to present substantial clinical challenges. LARC refers to RC characterized by tumor infiltration into surrounding tissues (T3–T4) or involvement of regional lymph nodes (N+) in the absence of distant metastasis (M0). The conventional standard therapeutic strategy involves neoadjuvant chemoradiotherapy (nCRT) followed by total mesorectal excision (TME), which has been shown to effectively downstage tumors, diminish the occurrence of positive surgical margins, and enhance both R0 resection rates and sphincter preservation in low RC cases (2). Nonetheless, the pathological complete response (pCR) rate remains limited to 10%–20%, and approximately 30% of patients continue to face a high risk of distant metastasis (3). Microsatellite instability-high (MSI-H), resulting from deficient mismatch repair (dMMR), appears in approximately 2.7% of RC cases (4), whereas the majority of RCs exhibit the proficient mismatch repair (pMMR)/microsatellite stable (MSS) phenotype. Immune checkpoint inhibitors (ICIs), a pivotal modality in cancer immunotherapy, have achieved significant breakthroughs in treating diverse malignant neoplasms (5). The ICIs currently in clinical use primarily include inhibitors targeting Programmed Cell Death Protein 1 (PD-1)/Programmed Death-Ligand 1 (PD-L1) and Cytotoxic T-Lymphocyte-Associated Protein 4 (CTLA-4). PD-1, located on the surface of T cells, bonds with its binding partner PD-L1, which is found in cancer cells, consequently resulting in T cell exhaustion, inhibiting activation and cytolytic function, and promoting the conversion of effector T cells to regulatory T cells (6–8). Anti-PD-1 antibodies (e.g., nivolumab, pembrolizumab) and anti-PD-L1 antibodies (e.g., atezolizumab, durvalumab) mitigate this suppression, thereby enhancing T cell-mediated immune responses (9). CTLA-4 functions as an immune checkpoint that impedes the early activation of T cells through its interaction with B7 molecules (CD80/CD86). Anti-CTLA-4 antibodies (e.g., ipilimumab) interrupt this pathway, thereby promoting T cell activation and augmenting immune responsiveness (10). The tumor immune microenvironment (TIME) plays a pivotal role in determining a tumor’s responsiveness to immunotherapy. Based on the density and spatial distribution of tumor-infiltrating lymphocyte (TIL), TIME can be categorized into four immune phenotypes (11) (1): “hot” tumors, characterized by abundant TIL and frequent T-cell dysfunction or exhaustion, often accompanied by activation of immune checkpoints such as PD-1, CTLA-4, T-cell immunoglobulin and mucin-domain containing-3 (TIM-3), and lymphocyte-activation gene 3 (LAG-3) (2); altered–immunosuppressed tumors, which exhibit reduced but not absent T-cell infiltration, along with enrichment of immunosuppressive cells such as myeloid-derived suppressor cells (MDSCs) and regulatory T cells (Tregs), as well as increased levels of soluble inhibitory mediators including transforming growth factor-beta (TGF-β), interleukin-10 (IL-10), and vascular endothelial growth factor (VEGF), and immune checkpoint expression, indicating functional suppression (3); altered–excluded tumors, where T cells accumulate at the invasive margin but fail to infiltrate the tumor core, typically due to oncogenic signaling, epigenetic dysregulation, aberrant vasculature, dense stromal barriers, and hypoxia; and (4) “cold” tumors, defined by a lack of T-cell infiltration within both the tumor core and its margins, often due to defective antigen presentation, low tumor mutational burden, or intrinsic resistance to cytotoxic T-cell killing; Clinically, pMMR/MSS rectal cancers are often classified as “cold” or “altered–excluded” phenotypes, marked by limited immune cell infiltration and multiple immune evasion mechanisms, such as low tumor mutational burden (TMB) and the presence of immunosuppressive cell populations (12, 13). The advent of ICIs, encompassing PD-1/PD-L1 and CTLA-4 inhibitors, has introduced novel therapeutic avenues for managing solid tumors, such as RC. In particular, treatment with dostarlimab in dMMR/MSI-H RC patients has resulted in a clinical complete response (cCR) rate of 100% (14), positioning immunotherapy as the recommended modality for dMMR/MSI-H LARC. Immune cell infiltration has been identified as a favorable prognostic marker in RC, independent of tumor stage or mismatch repair status (15, 16). In contrast, pMMR/MSS-type RC is typically characterized by reduced immune cell infiltration and a lower mutational burden in tumor-associated antigens (17). Consequently, clinical trials involving monotherapy with ICIs in this subgroup have demonstrated unsatisfactory efficacy. Combination regimens incorporating anti-PD-1 or anti-PD-L1 antibodies with anti-CTLA-4 antibodies (18), tyrosine kinase inhibitors, or small-molecule agents (19, 20) have similarly failed to yield anticipated therapeutic benefits. To address this therapeutic challenge, multiple emerging combination strategies are being actively explored to enhance antitumor immune responses, including the use of radiotherapy (administered as either long course or short course schedules), cytotoxic chemotherapy (e.g., CAPOX or mFOLFOX regimens), and anti-angiogenic tyrosine kinase inhibitors (e.g., fruquintinib) in combination with immune checkpoint inhibitors.

In recent years, the therapeutic strategy involving the combination of ICIs with nCRT, particularly in the context of patients diagnosed with pMMR/MSS-type LARC, has progressively become a focal point of academic inquiry. This review seeks to provide a comprehensive synthesis assessment of the existing research on the integration of ICIs with nCRT in pMMR/MSS-type LARC while further examining the underlying mechanisms, therapeutic efficacy, and the investigation of associated biomarkers relevant to this combinatorial treatment paradigm.

## 2 Theoretical basis for combining nCRT with immunotherapy

### 2.1 Chemotherapy combined with immunotherapy

Cold tumors are typically characterized by limited responsiveness to immunotherapy, primarily due to inadequate infiltration of immune cells. However, evidence has demonstrated that certain chemotherapeutic agents, including fluorouracil, oxaliplatin, and irinotecan, not only suppress tumor progression through direct cytotoxic effects but also potentiate immune responses within the tumor microenvironment (TME) via multiple mechanisms. These agents are demonstrated to stimulate the immune system and improve tumor sensitivity to ICIs by inducing immunogenic cell death, enabling the release of tumor-associated antigens (TAA), and elevating PD-L1 expression on tumor cells (21–23). In the context of pMMR/MSS RC cold tumors, the combinatorial use of chemotherapy and immunotherapy has been extensively investigated. For instance, several studies have reported that simultaneously administering chemotherapy and ICIs (e.g., PD-1/PD-L1 inhibitors) augments antitumor immunity by increasing immune cell infiltration within the tumor immune microenvironment. Furthermore, triple-combination therapy—comprising chemotherapy, immunotherapy, and anti-vascular endothelial growth factor agents—has exhibited synergistic effects against pMMR/MSS cold tumors, effectively modulating the TME and enhancing the therapeutic efficacy of immunotherapy (24, 25).

### 2.2 Radiotherapy combined with immunotherapy

**Figure 1:**
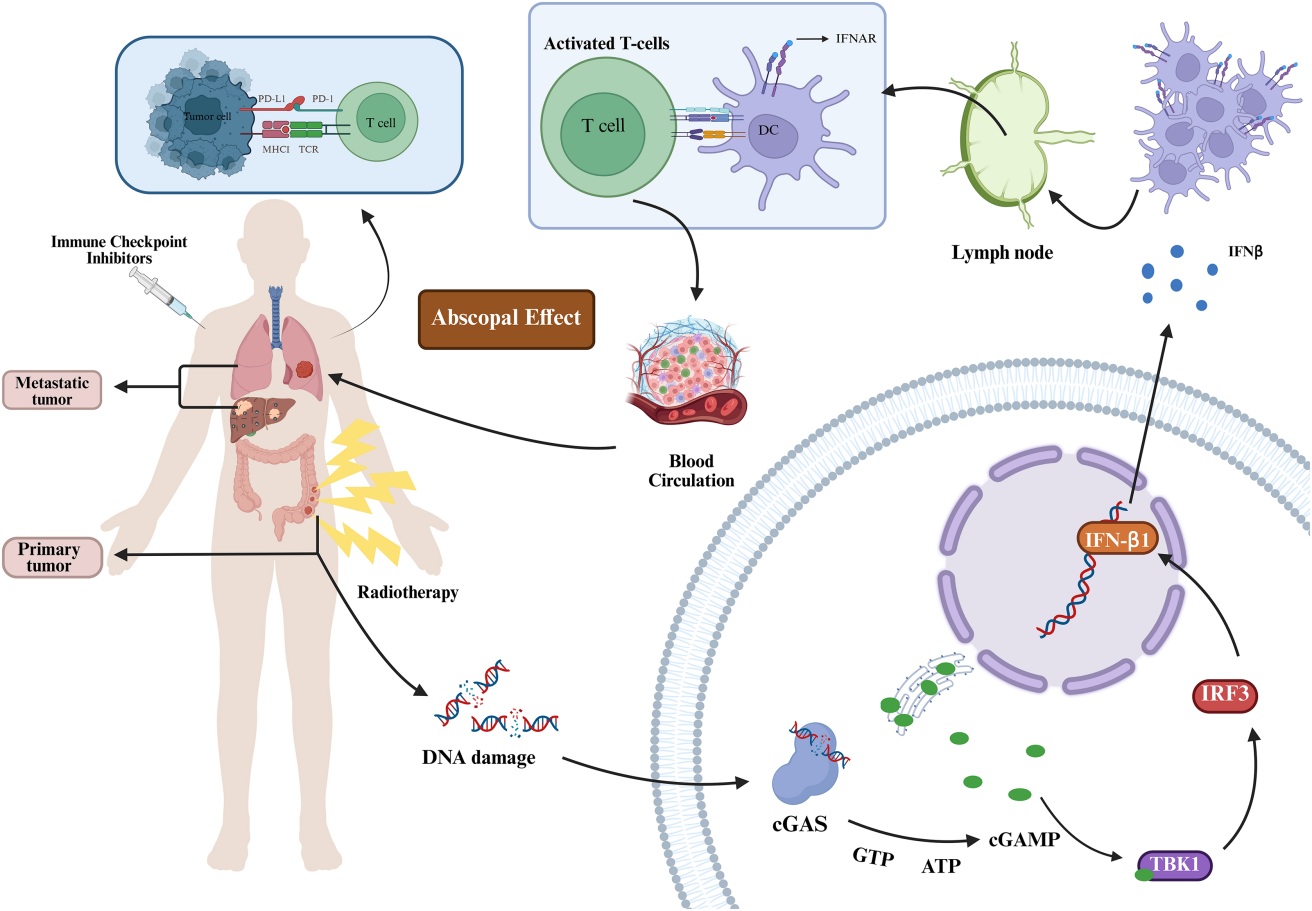
Immune priming and the abscopal effect induced by radiotherapy. Radiotherapy induces DNA double-strand breaks in tumor cells, leading to the release of DNA fragments into the cytoplasm, where they are detected by cyclic GMP–AMP synthase (cGAS). Upon binding cytosolic DNA, cGAS is activated to catalyze the synthesis of cyclic GMP–AMP (cGAMP), which acts as a second messenger to activate stimulator of interferon genes (STING) located on the endoplasmic reticulum membrane. Activated STING recruits and phosphorylates TANK-binding kinase 1 (TBK1), subsequently activating interferon regulatory factor 3 (IRF3). Phosphorylated IRF3 translocates to the nucleus and initiates the transcription of type I interferons (e.g., IFN-b) and inflammatory cytokines, enhancing dendritic cell (DC) function and promoting CD8+ T cell activation as well as MHC-I upregulation. These activated tumor-specific T cells proliferate and migrate to distant, non-irradiated tumor sites, mediating cytotoxicity and inducing the abscopal effect. Immune checkpoint inhibitors block inhibitory signaling pathways such as PD-1/PD-L1, relieving T cell exhaustion and amplifying both local and systemic antitumor immune responses.

**Figure 2:**
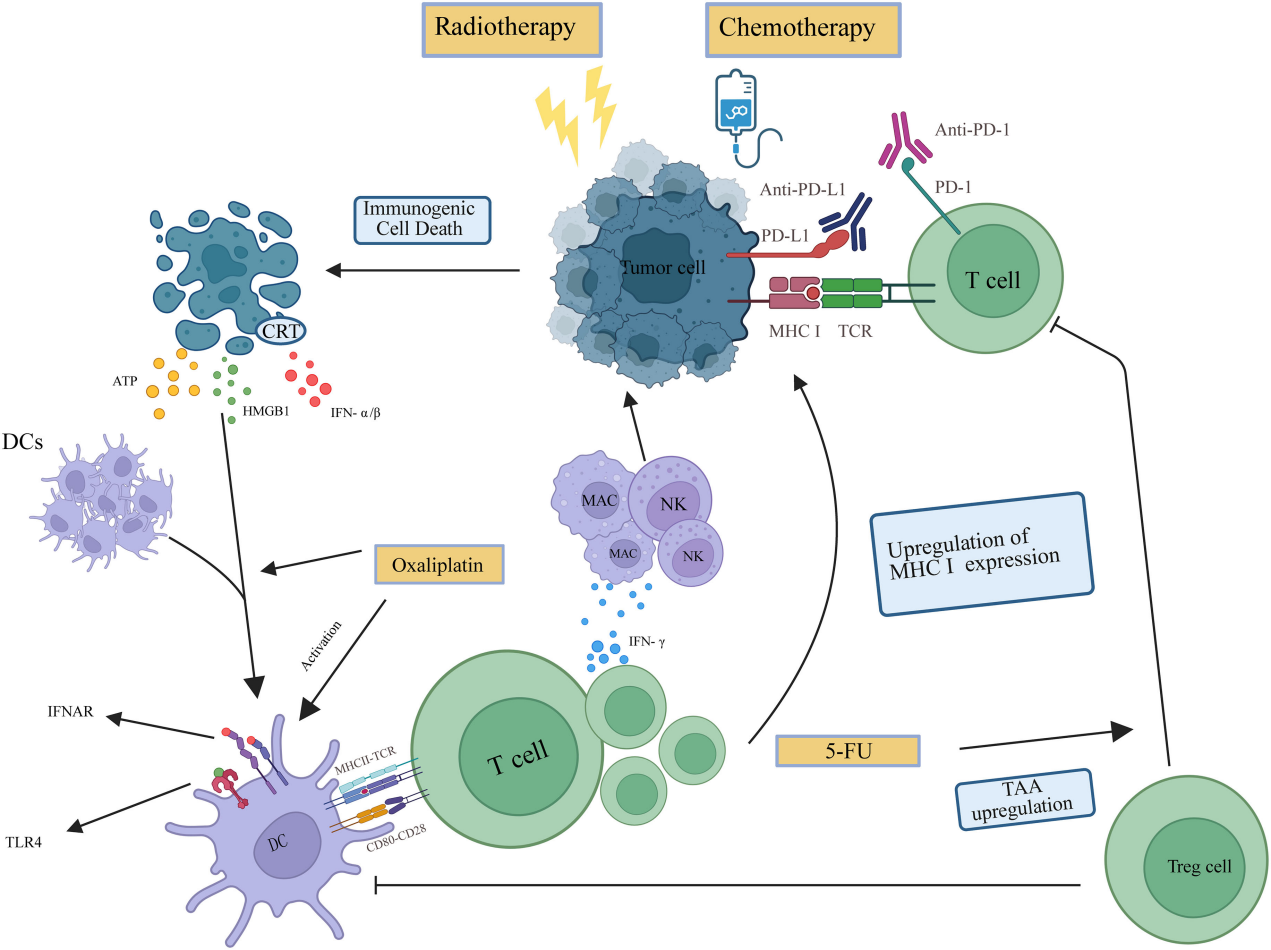
The underlying mechanisms for combining nCRT with immunotherapy. Radiotherapy or certain chemotherapeutic agents can induce immunogenic cell death (ICD) in tumor cells, characterized by three key damage-associated molecular pattern (DAMP) signals (1): surface exposure of calreticulin (CRT) triggered by endoplasmic reticulum stress, which facilitates dendritic cell (DC) recognition and phagocytosis (2); active release of ATP, which serves as a chemoattractant to recruit immune cells and activate DCs; and (3) release of high-mobility group box 1 (HMGB1) upon cell death, which binds to TLR4 on DCs to further enhance antigen presentation. DCs present tumor antigens to T cells via MHC molecules and secrete IFN-g, which in turn activates macrophages and natural killer (NK) cells, enhances antigen presentation, and upregulates MHC-I expression to promote cytotoxic T lymphocyte (CTL) function. 5-fluorouracil increases tumor-associated antigens (TAAs) and reduces Treg-mediated suppression of DCs and T cells. Oxaliplatin specifically induces ICD. PD-L1 blockade relieves T-cell functional exhaustion by inhibiting the interaction with its receptor on T cells.

In investigations concerning the immunomodulatory effects of radiation therapy, radiotherapy has been shown to exert immune-stimulating actions within the TME through three principal mechanisms (1): induction of immunogenic cell death (ICD) in tumor cells; (2) upregulation of neoantigen presentation via major histocompatibility complex class I molecules; and (3) direct modulation of the TME at the site of irradiation (26). ICD leads to the release of key damage-associated molecular patterns (DAMPs), including ATP, HMGB1, and calreticulin, which collectively facilitate the recruitment and maturation of dendritic cells (DCs). Concurrently, activation of the cyclic GMP-AMP synthase-stimulator of interferon genes (cGAS–STING) pathway triggers a type I interferon (IFN-I) response, further enhancing the functional activation of DCs. These activated DCs subsequently migrate to draining lymph nodes, where they efficiently cross-present TAA and prime tumor-specific CD8^+^ cytotoxic T lymphocytes, ultimately initiating a robust adaptive immune response (26, 27). This immune priming mechanism serves as a fundamental basis for eliciting a systemic antitumor response. Activated tumor-specific T cells can proliferate *in vivo* and migrate to distant, non-irradiated tumor sites, where they mediate cytotoxic activity against tumor cells—an effect referred to as the “abscopal effect” (28, 29) (**Figure 1**). Moreover, radiotherapy has been demonstrated to augment surface molecule levels on tumor cells, consequently enhancing recognition and cytotoxic activity by T cells and natural killer cells. Beyond the irradiated field, systemic immune responses may be elicited through abscopal effects—a phenomenon substantiated by multiple studies. The immune-enhancing properties of radiotherapy thus provide a compelling mechanistic rationale for its integration with PD-1/PD-L1 inhibitors, particularly in pMMR/MSS LARC patients who exhibit resistance to PD-1/PD-L1 monotherapy (**Figure 2**).

## 3 Advances in clinical trials of nCRT combined with immunotherapy

In recent years, multiple investigations have suggested that radiotherapy markedly elevates the antitumor efficacy of ICIs (30, 31). Among pMMR/MSS LARC patients who undergo neoadjuvant radiotherapy, the potential presence of a radiosensitizing effect that could elevate the pCR rate has been investigated in several studies, and preliminary findings have been reported (**Tables 1**–**3**). Depending on the duration and structure of radiotherapy regimens, current clinical practice generally categorizes combined neoadjuvant and immunotherapy strategies into two primary models: long-course chemoradiotherapy (LCRT) and short-course radiotherapy (SCRT). Moreover, total neoadjuvant therapy (TNT) combined with immunotherapy is increasingly being incorporated into clinical applications.

**Table 1:** Clinical trial of long-course radiochemotherapy combined with immune checkpoint inhibitors for locally advanced rectal cancer.

Study	Phase	Sample sizes	Baseline features^a^	Trial design^b^	Results	Adverse effects^c^
Voltage-A	Ib/II	39	MSS	50.4Gy/Cape + Nivolumab*5 + TME + mFOLFOX6/CAPOX	pCR 30% cCR 28.5% IQR 56.4 mo 3-year RFS 79.5% 3-year OS 97.4%	G3–4 iAE: 3 cases
NSABP FR-2	II	45	MSS	LCRT + Durvalumab*4 + TME	mNAR 12.03 pCR 22.2% cCR 31.1% R0 81.0%	Most common G3: diarrhea, lymphopenia, and back pain G4: 1 case of elevation of Amylase/Lipase
PANDORA	II	50	MSS/MSI-H	50.4Gy/Cape + Durvalumab*3 + TME	pCR 32.7% Near-pCR 25.5% IQR 22.2 mo	G3: 7.3% G4: none
R-IMMUNE	Ib/II	39	III 82% MSI-H 12%	Arm1: 45–50 Gy/5-Fu/Atezolizumab*4 + TME Arm2: 45–50 Gy/5-Fu + TME	pCR 27%	G3-4: 49% G4 iAE: 1 case
BFH-NCRTPD	II	26	MSS	50Gy/Cape/Tislelizumab*3 + TME	pCR 50% Anus Preservation Rate 88.5%	G3 iAE: 3.8%
NECTAR	II	50	MSS 98%	50Gy/Cape/Tislelizumab*3 + TME	pCR 40% R0 100%	G3: 2 cases
POLARSTAR	II	171	MSS 88.3%	Arm1: 45-50.4Gy/Cape/Tislelizumab*3 + TME Arm2: 45-50.4Gy/Cape + Tislelizumab*3 + TME Arm3: 45-50.4Gy/Cape + TME	pCR Arm1: 27.1% Arm2: 32.1% Arm3: 14%	IR Arm1: 2% Arm2: 4% Arm3: 5% G3-4 Arm1: 3% Arm2: 5% Arm3: none
Chongqing	retrospective	62	MSS 96.8%	Arm1: 50.4Gy/Cape/anti-PD-1*2 + (anti-PD-1 + XELOX)*2 + TME Arm2: 50.4Gy/Cape/+ (anti-PD-1 + XELOX)*2 + TME Arm3: 5*5Gy + (anti-PD-1 + XELOX)*2 + TME Arm4: (anti-PD-1 + XELOX)*2 + 5*5Gy + TME	pCR MSS 43.3% Anus Preservation Rate 88.7%	Most common: radiation enteritis 74.2%

a. “MSS” indicates that the study either exclusively enrolled patients with MSS tumors or included a specific subgroup analysis of MSS patients. “MSS/MSI-H” indicates that the study population was not stratified by microsatellite status.

b. “/” indicates that immunotherapy was administered concurrently or with overlapping timing, while “+” indicates that immunotherapy was initiated sequentially, following the completion of the preceding treatment modality. TME: Total Mesorectal Excision, a standard surgical procedure for rectal cancer.

c. G3/G4 refers to grade 3 or 4 adverse events, while irAE indicates immune-related adverse events. IR: the incidence rates of disease progression. IRR: an increased local recurrence rate.

**Table 2:** Clinical trial of short-course radiotherapy combined with immune checkpoint inhibitors for locally advanced rectal cancer.

Study	Phase	Sample sizes	Baseline features^a^	Trial design^b^	Results	Adverse effects^c^
Chengdu	II	23	MSS	(Fruquintinib + Toripalimab)*4/5*5Gy + TME	pCR 37.5% R0 100% Anus Preservation Rate 75%	G4-5: none
Wuhan	II	26	MSS	5*5Gy + (CAPOX + Camrelizumab)*2 + TME	pCR 46.2%	G3–4 iAE: none
UNION	III	231	MSS 94% stage III 82.3%	Arm1: 5*5Gy + (CAPOX + Camrelizumab)*2 + TME + (CAPOX + Camrelizumab)*6 Arm2: 50.4Gy/cape + TME + CAPOX*6	pCR Arm1: 39.8% Arm2: 15.3%	G3 Arm1: 16.8% Arm2: 17.5%
STELLAR II	II/III	588	/	Arm1: 5*5Gy + (Sintilimab + CAPOX)*4 or (Sintilimab*4 + mFOLFOX*6) + TME/W&W Arm2: 5*5Gy + CAPOX*4/mFOLFOX*6 + TME/W&W	pCR cCR	/
Shanghai	II	50	/	5*5Gy + (Cadonilimab + CAPOX)*4/(Cadonilimab*4 + mFOLFOX*6) + TME	pCR MPR	/
Chongqing	retrospective	62	MSS 96.8%	Arm1: 50.4Gy/Cape/anti-PD-1*2 + (anti-PD-1 + XELOX)*2 + TME Arm2: 50.4Gy/Cape/+ (anti-PD-1 + XELOX)*2 + TME Arm3: 5*5Gy + (anti-PD-1 + XELOX)*2 + TME Arm4: (anti-PD-1 + XELOX)*2 + 5*5Gy + TME	pCR MSS 43.3% Anus Preservation Rate 88.7%	Most common: radiation enteritis 74.2%

a. “MSS” indicates that the study either exclusively enrolled patients with MSS tumors or included a specific subgroup analysis of MSS patients. “MSS/MSI-H” indicates that the study population was not stratified by microsatellite status.

b. “/” indicates that immunotherapy was administered concurrently or with overlapping timing, while “+” indicates that immunotherapy was initiated sequentially, following the completion of the preceding treatment modality. TME: Total Mesorectal Excision, a standard surgical procedure for rectal cancer.

c. G3/G4 refers to grade 3 or 4 adverse events, while irAE indicates immune-related adverse events. IR: the incidence rates of disease progression. IRR: an increased local recurrence rate.

**Table 3:** Clinical trial of total neoadjuvant therapy combined with immune checkpoint inhibitors (ICIs) for locally advanced rectal cancer.

Study	Phase	Sample sizes	Baseline features^a^	Trial design^b^	Results	Adverse effects^c^
Averectal	II	44	MSS	5*5Gy + (mFOLFOX6 + Avelumab)*6 + TME	pCR 37.5% MPR 67.5% IQR 44 mo 3-year DFS 85%	G3–4 iAE: 1 case IRR 2.5% Death (progression): 7.5%(3/40)
NRG-G1002	II	185	MSS/MSI-H	Arm1: mFOLFOX6*8 + 50.4Gy/Cape + TME Arm2: mFOLFOX6*8 + 50.4Gy/Cape/Pembrolizumab + TME	NAR Arm1: 14.08 Arm2: 11.53 pCR Arm1: 29.4% Arm2: 31.9%	G3-4: none
PKUCH04	II	25	MSS	(CAPOX + Camerelizumab)*3 + 45Gy/Cape + CAPOX*2 + TME	pCR 33.3% MPR 71.4%	G3: lymphopenia 24%, diarrhea 8%, thrombocytopenia 4%
ESTIMATE	II	28	MSS	(mFOLFOX6 + Envafolimab)*3 + 50Gy/Cape/Envafolimab*3 + (mFOLFOX6 + Envafolimab)*2 + TME/W&W	cCR 42.9% pCR 38.1% MPR 71.4% IQR 5 mo	G3: 7.1% (primarily leukopenia or neutrophilia)
TORCH	II	130	MSS 93.1%	Arm1: SCRT + (CAPOX + Toripalimab)*6 + TME/W&W Arm2: (CAPOX + Toripalimab)*2 + SCRT + (CAPOX + Toripalimab)*4 + TME/W&W	CR Arm1: 56.5% Arm2: 54.2% pCR both 50%	G3-4 Arm1: 45.2% Arm2: 42.4%

a. “MSS” indicates that the study either exclusively enrolled patients with MSS tumors or included a specific subgroup analysis of MSS patients. “MSS/MSI-H” indicates that the study population was not stratified by microsatellite status.

b. “/” indicates that immunotherapy was administered concurrently or with overlapping timing, while “+” indicates that immunotherapy was initiated sequentially, following the completion of the preceding treatment modality. TME: Total Mesorectal Excision, a standard surgical procedure for rectal cancer.

c. G3/G4 refers to grade 3 or 4 adverse events, while irAE indicates immune-related adverse events. IR: the incidence rates of disease progression. IRR: an increased local recurrence rate.

### 3.1 Research progress of ICIs combined with LCRT

LCRT is primarily administered with immunotherapy via sequential or concurrent approaches (**Table 1**). In sequential immunotherapy following LCRT, several Phase II, single-arm, non-randomized clinical trials—such as VOLTAGE-A, NSABP FR-2, and PANDORA—have assessed its therapeutic efficacy, all of which have reported promising pCR rates. Specifically, the Japanese VOLTAGE-A study (32) implemented a regimen of nCRT succeeded by five cycles of nivolumab monotherapy and subsequent TME surgery. This trial enrolled 39 patients with pMMR/MSS LARC; among them, 37 were included in the primary endpoint analysis, and all participants were evaluated for both efficacy and safety. The findings demonstrated a pCR rate of 30% (11/37; 90% confidence interval [CI], 18%–44%). Regarding safety, only three patients experienced adverse events in grades III–IV. The VOLTAGE-A study (33) later reported long-term follow-up results in 2024, with a median (interquartile range) observation period of 56.4 (51.6–57.6) months. Among pMMR/MSS LARC patients, the 3-year relapse-free survival rate reached 79.5% (95% CI, 63.1%–89.2%), while the 3-year overall survival (OS) was 97.4% (95% CI, 83.2%–99.6%). During the post-TME observation period, eight cases of tumor recurrence were recorded in the pMMR/MSS LARC cohort. In addition, two deaths occurred at 33 and 38 months following completion of chemoradiotherapy (CRT) in individuals with MSS LARC who experienced cancer recurrence. The study results showed a trend toward improved 3-year recurrence-free survival and overall survival in patients who achieved pCR.

The NSABP FR-2 study incorporated 45 individuals diagnosed with pMMR/MSS stage II–IV RC. The treatment protocol involved CRT, which was succeeded by four cycles of durvalumab immunotherapy, with the median modified neoadjuvant rectal (mNAR) Score designated as the primary endpoint. Among the 40 evaluable patients, the mean mNAR was reported as 12.03 (80% CI: 9.29–14.97, *p* = 0.06). A pCR rate of 22.2% was observed, with only one instance of a grade IV adverse reaction (elevated amylase/lipase) documented (34). The Italian PANDORA study (35) included 55 individuals with LARC who received three cycles of durvalumab monotherapy following nCRT. Results demonstrated that 34.5% of patients (19/55) achieved pCR(95% CI, 22.2%-48.6%). Furthermore, the incidence of grade III–IV toxicity related to either nCRT or durvalumab remained low. However, The absence of stratification by microsatellite status (MSS vs. MSI-H) in the PANDORA study may have contributed to a potentially inflated pCR rate. The study reported a median follow-up duration of 22.2 months (95% CI, 20.2–26.1 months), and the median disease-free survival (DFS) was not reached. In comparison to the conventional pCR rate of approximately 15%–20% typically achieved with standard nCRT, all three aforementioned studies reported pCR rates exceeding 30%, with overall complete response (CR) rates also surpassing this range, suggesting a substantial enhancement in tumor response through the integration of nCRT and immunotherapy. Different trials showed varying pCR rates. The PANDORA study demonstrated a higher pCR rate compared to the NSABP FR-2 trial, which may be related to a longer interval between the end of radiotherapy and surgery. In the VOLTAGE-A study, preoperative screening revealed higher pCR rates in patients with PD-L1 TPS ≥ 1% and a CD8+/eTreg ratio ≥ 2.5 (67% vs. 17% and 78% vs. 13%, respectively). In contrast, the NSABP FR-2 trial did not specifically analyze such biomarkers and enrolled patients based on conventional MSS status, possibly including more immunologically low-responsive patients.

In recent years, the efficacy of LCRT in combination with immunotherapy has been examined in multiple studies, yielding varying clinical outcomes. R-IMMUNE (36), a multicenter Phase Ib/II clinical trial, sought to assess the effectiveness and safety of CRT in conjunction with atezolizumab for individuals with stage II/III LARC. In Phase Ib component (37), a single infusion of atezolizumab was administered at week 3, whereas the Phase II portion involved four infusions at weeks 3, 6, 9, and 12. A sum of six patients were enrolled in the Phase Ib cohort, while 34 patients participated in the Phase II study. Among the 39 patients evaluated, 12% were identified as MSI-H. One patient in the Phase II cohort who remained under treatment was excluded from the final analysis. Grade III–IV adverse events were reported in 19 of 39 patients (49%). Two additional patients were excluded due to protocol non-adherence. Ultimately, a pCR rate of 27% (10/37), including two MSI-H patients, was achieved.

The BFH-NCRTPD is a prospective, multicenter, single-arm, Phase II study (38), assessed the concurrent administration of CRT and three cycles of tislelizumab in 26 patients, with treatment completed between April 2021 and June 2022. Interim findings demonstrated that 50% of pMMR/MSS patients (13/26) achieved pCR, while the sphincter preservation rate reached 88.5% (23/26). Only one patient experienced a grade III adverse event. In addition, the study investigated the correlation between carcinoembryonic antigen (CEA) levels and pCR outcomes, revealing that patients under 50 years of age without CEA elevation exhibited a pCR rate of 70.6% (12/17), in contrast to a rate of only 11.1% (1/9) among those with elevated CEA levels (*p* = 0.004). These results suggest that CRT combined with immunotherapy elicited a pronounced tumor response in patients with LARC, with CR and objective response rate (ORR) reaching 46.2% and 73.1%, respectively. Further enhancement of CR rates is of considerable clinical relevance for facilitating “Watch and Wait” (W&W) strategies or selective local excision in the pursuit of organ preservation.

The NECTARis a Phase II, multicenter, prospective, single-arm study (39), enrolled 50 patients, among whom 49 were classified as having the pMMR/MSS subtype. The findings indicated an overall pCR rate of 40.0% (20/50; 95% CI: 27.61%–53.82%). Tumor regression was documented in 52% of cases, while 56% of patients with LARC experienced treatment-related adverse events (TRAEs), including 26 instances (52%) of grade I–II and two instances (4%) of grade III adverse events—specifically, one case of grade III immune-related colitis and one case of grade III rash. Radical surgery was performed in all 46 patients, with an R0 resection rate of 100% and a sphincter preservation rate of 89.1% (41/46). The BFH-NCRTPD and NECTAR studies provide valuable references for organ preservation therapy.

In the recent randomized controlled Phase II study by Yang et al. (40), the efficacy of nCRT with or without PD-1 inhibitors was compared, and the potential differences in outcomes between synchronous and sequential immunotherapy were assessed. A total of 186 eligible patients—including both pMMR/MSS and dMMR/MSI-H subtypes—were enrolled and randomly assigned to receive nCRT in combination with either synchronous or sequential PD-1 inhibitors (Experimental Groups A and B) or nCRT alone (control group). Radical surgery was scheduled for all patients following neoadjuvant therapy. Fifteen patients were excluded due to failure to initiate the designated treatment. The final sample included 59, 55, and 57 patients in Experimental Groups A, B, and the control group, respectively, with the majority being pMMR/MSS cases (54, 48, and 49 patients, respectively). The pCR rates for synchronous and sequential immunotherapy were 27.1% and 32.7%, respectively, compared to 14% in the control group. A statistically significant difference was detected between sequential treatment and the control group (hazard ratio: 2.332; 95% CI: 1.106–4.916; *p* = 0.019). Regarding sphincter preservation rates of 88%, 87%, and 70% were achieved in Groups A, B, and the control group, respectively, while R0 resection rates were 97%, 91%, and 77%, respectively. Moreover, no substantial differences were identified among the groups with respect to adverse events, disease progression, or surgical complications. These findings suggest that incorporating PD-1 inhibitors with nCRT substantially improved pCR rates in individuals with LARC without introducing additional safety concerns. Notably, this trial was the first to implement distinct timing protocols for PD-1 inhibitor administration, with Group A receiving treatment on day 8 after radiotherapy and Group B two weeks following radiotherapy in an effort to optimize immunotherapy timing by leveraging radiation-induced microenvironmental changes. The sequential regimen was associated with significantly higher pCR rates than the synchronous regimen, potentially attributable to the complete remodeling of the tumor microenvironment after the conclusion of radiotherapy.

### 3.2 Research progress of ICIs combined with SCRT

Multiple studies have substantiated the potential of neoadjuvant SCRT in combination with immunotherapy for treating LARC, particularly demonstrating favorable outcomes in improving pCR rates, extending progression-free survival (PFS), and facilitating organ preservation (**Table 2**). In a prospective, single-arm, Phase II study (41) conducted in Chengdu, Sichuan, the efficacy of fruquintinib (an anti-angiogenic agent) and toripalimab (a PD-1 inhibitor), administered in conjunction with SCRT as neoadjuvant therapy, was investigated. Based on Simon’s two-stage design, the trial aimed to enroll 40 patients; as of February 2024, 23 had been recruited. Among them, nine patients completed neoadjuvant therapy, and 16 underwent TME surgery, resulting in an R0 resection rate of 100% and a sphincter preservation rate of 75% (12/16). Six patients achieved pCR, corresponding to a pCR rate of 37.5% (95% CI: 13.8%–61.2%). Notably, no grade IV or V TRAEs were observed. This study remains ongoing, and future data are anticipated to further support the efficacy and safety of this neoadjuvant regimen. A Phase II single-arm clinical trial (42) conducted by LIN et al. evaluated SCRT in combination with chemotherapy and camrelizumab (a PD-1 inhibitor) as preoperative therapy for LARC. The findings revealed a pCR rate of 46.2% (12/26) in pMMR/MSS patients, and the treatment exhibited acceptable safety and tolerability. On the basis of these promising outcomes, the multicenter, randomized Phase III UNION trial (43) was subsequently initiated to further examine the efficacy and safety of SCRT + camrelizumab + chemotherapy versus LCRT + adjuvant chemotherapy as neoadjuvant strategies for LARC. Subjects diagnosed with T3–4/N+ rectal adenocarcinoma underwent random allocation (1:1) to receive either SCRT or LCRT, subsequently followed by two cycles of camrelizumab and capecitabine plus oxaliplatin (CAPOX) or CAPOX alone, respectively. Following surgery, both groups were administered six cycles of camrelizumab and CAPOX, succeeded by either a maximum of 17 additional doses of camrelizumab or six extra cycles of CAPOX. During the period from July 2021 to March 2023, the study enrolled a total of 113 subjects in the experimental arm and 118 in the control arm. The experimental group exhibited a pCR rate of 39.8% (95% CI: 30.7%–49.5%) versus 15.3% (95% CI: 9.3%–23.0%) in the control group (difference: 24.6%; odds ratio: 3.7; 95% CI: 2.0–6.9; *p* < 0.001). Surgical complications occurred at frequencies of 40.0% and 40.8%, while grade III or higher TRAEs were documented in 29.2% and 27.2% of participants in the experimental and control cohorts, respectively. The pCR rate was the highest observed among all SCRT regimens in Phase III trials for LARC, and the incidence of grade 3 toxicities (16.8%) was also lower than those observed in other SCRT followed by chemotherapy treatments, including 47.6% in the RAPIDO trial, 26.5% in the STELLAR trial, and 24.2% in the Polish Phase II trial (44–46).

The STELLAR II study (47) is an ongoing multicenter, open-label, two-arm, randomized Phase II/III trial conducted in China. The study aims to include 588 individuals diagnosed with LARC, who will be allocated randomly between iTNT and TNT treatment protocols. The selected participants in both arms will receive SCRT (25 Gy/5 Fx), after which they will undergo either four rounds of CAPOX or six rounds of mFOLFOX chemotherapy, while the iTNT group will additionally incorporate sintilimab into their identical chemotherapy schedule. TME surgery is scheduled to follow the completion of neoadjuvant therapy. Key outcomes to be evaluated include patient CR rates, adverse events, and long-term prognosis. Recruitment is currently ongoing. This trial adopts a seamless Phase II/III randomized controlled design, thereby eliminating the need for a separate follow-up and data analysis phase after Phase II, and enabling more robust efficacy data generation within a shorter timeframe. Whether the integration of immunotherapy into the neoadjuvant regimen will lead to improved tumor regression, favorable tolerability, and enhanced prognosis remains to be determined. Improved clinical outcomes and the identification of optimal therapeutic strategies are anticipated. In addition, another ongoing Phase II, multicenter, single-arm, open-label, prospective trial from China (48) is evaluating SCRT combined with bispecific antibody immunotherapy and chemotherapy as a neoadjuvant regimen for patients with LARC. Preliminary data suggest that this combination demonstrates both efficacy and safety, and it may represent a viable candidate strategy for future LARC management.

A recent retrospective study (49) compared the efficacy of neoadjuvant LCRT or SCRT in combination with immunotherapy and assessed differences in outcomes across various treatment regimens. The study population comprised 62 LARC patients, among which 96.8% were classified as having the pMMR/MSS subtype, with only two patients identified as dMMR/MSI-H. Four distinct neoadjuvant protocols were evaluated: Protocol A (LCRT plus PD-1 inhibitor/capecitabine plus PD-1 inhibitor/CAPOX followed by TME), Protocol B (LCRT plus capecitabine plus PD-1 inhibitor/CAPOX succeeded by TME), Protocol C (SCRT plus PD-1 inhibitor/CAPOX followed by TME), and Protocol D (PD-1 inhibitor/CAPOX plus SCRT followed by TME). The findings demonstrated an overall pCR rate of 45.2%, with a pCR rate of 43.3% among pMMR/MSS patients. Across all regimens, tumor downstaging was achieved in 79% of cases, and the overall sphincter preservation rate was 88.7%. Among the four protocols, Protocol C exhibited the most favorable outcomes, with a tumor downstaging rate of 85.7% and a sphincter preservation rate of 100%. Protocol A ranked second, yielding an 85% downstaging rate and an 85% sphincter preservation rate. In contrast, Protocol D was associated with relatively lower tumor downstaging rates and sphincter preservation rates. The most frequently reported adverse event across the cohorts was radiation enteritis, with the highest incidence observed in Protocol A.

### 3.3 Research progress of ICIs combined with TNT

Suboptimal compliance with adjuvant therapy is one possible explanation for the elevated distant recurrence rate following nCRT. In fact, after completion of neoadjuvant treatment and surgery, only approximately 75% of patients initiate, and merely 50% complete, adjuvant chemotherapy (50). The TNT approach is designed to improve adherence to systemic chemotherapy, address micrometastases at an earlier stage, reduce the risk of distant recurrence, and enhance tumor response, thereby increasing the likelihood of organ preservation. Currently, TNT is primarily categorized into two strategies: induction chemotherapy followed by LCRT or consolidation chemotherapy administered after LCRT or SCRT (51). By shifting systemic therapy to the preoperative phase, TNT mitigates the historically low completion rates associated with postoperative chemotherapy and facilitates improved tumor regression (52).

In the 2020 open-label, single-arm, multicenter Phase II Averectal study (53), recruitment was planned for 44 patients. During the first phase, 13 patients with the pMMR/MSS subtype received SCRT in combination with six cycles of mFOLFOX6 chemotherapy and avelumab. Except for one patient who withdrew from the study prior to TME due to disease progression, all remaining participants completed the treatment protocol as scheduled. Results from the initial phase demonstrated that three patients (25%) achieved pCR. Final outcomes from the Averectal study (54) were presented at the 2024 ESMO GCC conference. Of the 44 patients enrolled, 40 completed the treatment and were included in the final analysis, with a median follow-up duration of 44 months (range: 11.4–51.4). Among these patients, 15 (37.5%) achieved pCR. The mean DFS was 42 months (range: 37.9–46.1), while the mean OS was 46.3 months (range: 44.4–48.2). However, median values for DFS and OS had not yet been reached. The 3-year DFS rate was 85%, and the local recurrence rate was 1 out of 40 patients (2.5%). Three patients died as a result of disease progression.

In 2021, the open-label, randomized, Phase II clinical NRG-G1002 study (55) reported on the efficacy of the pembrolizumab-containing regimen. This study compared treatment outcomes between a control group and an experimental group. The control group underwent eight cycles of FOLFOX chemotherapy succeeded by nCRT combined with capecitabine, whereas the experimental cohort received an identical protocol plus pembrolizumab. The investigation designated the NAR score as the principal endpoint. The findings revealed mean NAR scores of 14.08 and 11.53 for the control and pembrolizumab cohorts, respectively, showing no notable statistical variance (*p* = 0.26). Furthermore, the pCR rates measured 29.4% and 31.9% (*p* = 0.75), while cCR rates reached 13.6% and 13.9% (*p* = 0.95) in the control and pembrolizumab groups, respectively. Although statistical evaluation demonstrated similar tumor regression rates between both cohorts, the aggregate pCR and cCR rates approximated 44% in each, suggesting that approximately half of the patient’s achieved complete tumor response. This data indicates that incorporating TNT with immunotherapy might foster maximal tumor regression. Nevertheless, pembrolizumab supplementation failed to enhance tumor regression further, potentially due to the relatively low immunotherapy completion rate. Additionally, because radiotherapy exerts significant cytotoxic effects on lymphocytes, its concurrent administration with immunotherapy may impair immune efficacy by depleting locally accumulated or activated lymphocytes.

Another prospective, single-arm, non-randomized Phase II study, the PKUCH04 trial (56), conducted in Beijing, enrolled 25 high-risk patients with LARC. These patients received three cycles of CAPOX chemotherapy combined with camrelizumab, followed by LCRT and an additional two cycles of CAPOX chemotherapy, and subsequently underwent either TME or a W&W strategy. Among the 21 patients who proceeded to TME surgery, pCR was achieved in seven patients (33.3%), while a major partial response was observed in 15 patients (71.4%). The remaining four patients demonstrated clinical or near-complete clinical response following neoadjuvant treatment and were managed with the W&W approach. Additionally, its CR rate of 44% was higher than that observed in the NRG-GI002 study (31.9%) and the AVERECTAL study (37.5%). The primary grade III–IV adverse events included lymphocytopenia in 24% of patients, diarrhea in 8%, and thrombocytopenia in 4%, with no grade IV adverse reactions reported. This study indicated that the TNT regimen comprising nCRT in combination with immunotherapy significantly improved complete tumor response rates in patients with LARC, yielding markedly enhanced tumor regression compared to conventional therapy. The limitation of this study lies in the small number of enrolled patients and relatively short postoperative follow-up duration. Larger-scale prospective studies with long-term follow-up are needed to validate its efficacy and safety in pMMR/MSS LARC patients.

A prospective, single-arm, Phase II study from China, presented at the 2024 ASCO Annual Meeting (57), indicated that as of November 16, 2023, 35 patients had been enrolled, among whom 28 had completed the TNT regimen. Efficacy analysis suggested that, among these 28 patients, 12 (42.9%) achieved cCR, of whom seven chose the W&W strategy. Of the 21 patients who underwent TME surgery, tumor regression grades were as follows: grade 0 in eight patients, grade I in seven, and grade II in six. The pCR rate was 38.1% (8/21), the major pathologic response rate was 71.4% (15/21), and the CR rate was 53.6% (15/28). In addition, 89.3% of patients experienced at least a grade I TRAE, and six patients tested negative for minimal residual disease. The median follow-up period was five months (range: 3–6), with no recurrence detected in any patient. This study indicated that the TNT regimen—comprising LCRT, mFOLFOX6 chemotherapy, and a PD-L1 inhibitor—resulted in a favorable complete tumor response rate with acceptable tolerability, offering a potential treatment option for organ preservation in patients with pMMR/MSS low RC.

The Prospective, multicenter, randomized, Phase II TORCH study (58), published in the same year, employed a pick-the-winner design and enrolled eligible patients diagnosed with clinical T3–T4 and/or N1 stage rectal adenocarcinoma. Patients were randomized into Groups A and B. Group A received SCRT followed by six cycles of capecitabine, oxaliplatin, and toripalimab as consolidation immunochemotherapy, while Group B underwent two cycles of induction immunochemotherapy followed by SCRT, with subsequent assignment to either TME or the W&W strategy based on tumor response. Among the 130 enrolled patients, 121 were identified as having the pMMR/MSS subtype (62 in Group A and 59 in Group B). At a median follow-up of 19 months, the CR rates for Groups A and B were 56.5% and 54.2%, respectively, both significantly exceeding the predefined statistical hypothesis (*p* < 0.001). The pCR rate was 50% in both groups. Fifteen patients in each group selected the W&W strategy and remained disease-free. The most frequently reported grade III–IV adverse events included thrombocytopenia and neutropenia. Patients in Group A exhibited a higher cCR rate at restaging (43.5% vs. 35.6%) and a lower incidence of grade III–IV thrombocytopenia during neoadjuvant therapy (24.2% vs. 33.9%). The CR rates achieved in the TORCH trial were substantially higher than the pCR or CR rates reported in the experimental arms of the RAPIDO (44) or STELLAR (46) trials. The most plausible explanation currently proposed is that the addition of immunotherapy contributed to enhanced tumor regression.

Several studies have explored the incorporation of ICIs into TNT regimens, demonstrating encouraging pCR rates that surpass those observed in TNT protocols without immunotherapy, such as the RAPIDO study (pCR 28.4%) and the PRODIGE 23 study (pCR 27.5%). However, except for the NRG-GI002 trial, which included a randomized control arm with immunotherapy, the other studies were single-arm trials, limiting the ability to attribute observed efficacy specifically to the addition of ICIs. Furthermore, the NRG-GI002 trial showed no statistically significant difference in NAR scores between the experimental and control groups. Considering the high cost of immunotherapeutic agents and substantial resource utilization during treatment, the cost-effectiveness of incorporating ICIs into TNT regimens remains unsubstantiated in the absence of clear clinical benefits. Therefore, robust evidence from large-scale randomized controlled trials is essential to rigorously evaluate both the clinical efficacy and economic viability of adding ICIs to TNT protocols.

## 4 Predictive biomarkers

pMMR/MSS RC exhibits biological heterogeneity, thereby necessitating the identification of innovative prognostic biomarkers to determine patient populations most suitable for existing ICIs. Established biomarkers encompass tumor mutational burden (TMB) and PD-L1 expression levels.

TMB represents the number of somatic mutations per megabase in cancer cell DNA. Elevated TMB levels may result in the generation of neoantigens, which can subsequently activate immune responses, thereby potentially increasing tumor susceptibility to ICIs. Multiple studies have demonstrated that high TMB is associated with favorable responses to ICIs across diverse malignancies, supporting its value as a predictive biomarker (59). Nevertheless, data from the KEYNOTE-158 trial indicated that TMB-clinical benefit correlation varies among tumor types (60), possibly due to biological differences influencing antigen presentation pathways. Furthermore, in a Phase II study involving advanced pMMR/MSS Colorectal Cancer (CRC), high TMB was found to predict responses to anti-PD-L1 and CTLA-4 antibodies (18). However, among 5,702 sequenced MSS tumor samples, only 164 cases (2.9%) exhibited high TMB. Moreover, due to inconsistencies in detection methods, platforms, and threshold values (61–63), using TMB to identify potential benefit populations remains challenging. In contrast, in the NICHE-1 neoadjuvant trial, robust responses to ICIs were observed in pMMR/MSS patients despite their lower TMB levels (64). These findings suggest that the predictive value of TMB in the context of neoadjuvant immunotherapy warrants further investigation.

The tumor expression level of PD-L1 has been extensively investigated as a biomarker for predicting response to ICIs. Overexpression of PD-L1 has been associated with adverse clinicopathological features in patients with RC, including poor differentiation, lymphovascular invasion, and reduced OS (65). Although some evidence suggests that PD-L1 overexpression may be linked to unfavorable prognosis, its role as a predictive indicator of ICI efficacy remains a subject of ongoing debate. In the subgroup analysis of the IMblaze 370 study, no significant differences were observed between PD-L1 high and low groups in terms of OS, ORR, PFS, or OS rate in metastatic colorectal cancer (mCRC) (20).

Tumor-infiltrating lymphocyte (TIL) density refers to the number or proportion of lymphocytes infiltrating per unit area of tumor tissue. TIL density has been identified in previous studies as a predictive biomarker for treatment response and long-term prognosis in patients with LARC receiving nCRT (66, 67). High baseline CD8^+^ TIL density independently predicts increased pathologic complete response rates and prolonged survival (DFS/OS) in locally advanced rectal cancer. However, the assessment of TIL density using conventional H\&E staining largely relies on manual evaluation by pathologists, which introduces inter-observer variability and may affect the stability and reproducibility of the results. Furthermore, the lack of standardized clinical thresholds to define “high” or “low” TIL density limits its widespread implementation in clinical practice and treatment guidelines.

Modulation of the gut microbiome by altering microbial diversity can affect the efficacy of ICIs (68). Some studies (69) have found elevated levels of Fusobacterium nucleatum in the tumor tissue and fecal samples of CRC patients. The Phase II RENMIN-215 trial showed that in patients with treatment-refractory metastatic pMMR CRC, the combination of fecal microbiota transplantation (FMT), ICIs, and fruquintinib achieved a median OS of 13.7 months, outperforming similar studies without FMT (70, 71). These results suggest that the gut microbiome has potential as a predictive biomarker in CRC.

Immunoscore IC is an extension of the traditional Immunoscore, incorporating not only the density of CD3^+^ and CD8^+^ T cells but also their spatial relationship with PD-L1–expressing cells (24, 72). In the Phase II NICOLE trial (73), CD3^+^ and CD8^+^ T cell infiltration was significantly higher in patients with pathological stages 0–II compared to those with stage III disease (P = 0.029 and P = 0.044, respectively), although this infiltration was not correlated with pathological response. Similarly, the NICHE-1 study found that CD3^+^ and CD8^+^ T cell infiltration did not distinguish responders from non-responders in patients with pMMR CRC. Thus far, no neoadjuvant CRC trial has incorporated Immunoscore IC as a predictive biomarker, possibly due to limited availability of tissue samples at this treatment stage.

Genomic biomarkers reflect genetic alterations linked to disease susceptibility, prognosis, or therapeutic response. Circulating tumor DNA (ctDNA) can be detected in the plasma of both healthy individuals and patients with malignancies. In recent years, ctDNA has emerged as a promising prognostic and potentially predictive biomarker in the personalized management of patients with RC (74). Prior studies have demonstrated that ctDNA has been employed as a marker of chemotherapy response in patients with metastatic colorectal cancer (75–78). However, the application of ctDNA in assessing tumor response and prognosis in patients with LARC undergoing nCRT has only recently been explored (79–82). Preoperative baseline ctDNA testing has been shown to correlate with pathological TNM staging and tumor response. However, relying solely on ctDNA analysis to determine whether CR has been achieved remains limited. Therefore, at the current stage, it is not advisable to base the implementation of the W&W strategy entirely on ctDNA results. The long-term results of a recent Beijing-led prospective multicenter study (82) have demonstrated the potential of dynamic ctDNA monitoring as an actionable stratification biomarker for guiding personalized neoadjuvant treatment strategies. This study analyzed tissue and plasma samples from 103 patients, with plasma collected at the following time points: prior to chemoradiotherapy, two weeks after the initiation of nCRT, within 7 days before surgery, 7–14 days postoperatively, and annually for up to three years after surgery. The result revealed that those with a baseline ctDNA minimum variant allele frequency (mVAF) ≥0.5% or with detectable ctDNA two weeks after nCRT exhibited significantly poorer PFS and OS. Moreover, baseline mVAF and TMB, in combination with ctDNA clearance during nCRT, functioned as effective prognostic indicators capable of stratifying patients into high-risk and low-risk categories. The low-risk group—characterized by either undetectable ctDNA during nCRT with baseline mVAF <0.5% or undetectable ctDNA during nCRT with TMB ≥20/Mb—demonstrated significantly improved long-term survival outcomes. In the future, by integrating more sensitive ctDNA detection methods with genomic and epigenetic features, TME profiling, transcriptomic and radiomic data, the accuracy of CR assessment is expected to improve, thereby facilitating safer application of organ preservation strategies. In addition, detecting preoperative ctDNA and high-risk gene mutations has been widely recognized as useful for identifying patients unsuitable for the W&W strategy (83).

In addition to the aforementioned commonly studied immune therapy-related biomarkers, factors such as T-cell receptor beta variable, T-cell receptor beta joining genes, the T-cell co-stimulatory receptor CD226, the co-inhibitory receptor TIGIT, β2-microglobulin, and the gut microbiome have also been implicated in modulating immune responses and influencing the efficacy of immunotherapy (70, 71, 84–87). The successful clinical translation of these emerging biomarkers will require validation of their predictive accuracy through additional clinical trials, thereby facilitating their broader implementation in routine clinical practice.

## 5 Discussion on heterogeneity across studies

### 5.1 Optimal sequence between radiotherapy/chemotherapy and immunotherapy

The optimal sequencing of radiotherapy or chemotherapy with immunotherapy remains a subject of ongoing debate. In the retrospective study by Chen et al. (49), treatment sequencing was modified in Protocols C and D, in which SCRT and PD-1 inhibitors/XELOX were administered in varying orders. Based on the outcomes analysis, administering radiotherapy followed by immunotherapy and chemotherapy was associated with higher tumor downstaging and sphincter preservation rates. It has been hypothesized that radiotherapy may enhance the sensitivity of MSS-type LARC patients to immunotherapy. Prior studies have suggested a potential “mutual sensitization” effect between radiotherapy and immunotherapy (88, 89), implying that their combination may result in improved tumor regression and long-term therapeutic efficacy. In the TORCH study, patients were randomized into Group A (consolidation chemotherapy) and Group B (induction chemotherapy). At a median follow-up of 19 months, no statistically significant differences in CR or pCR rates were observed between the two groups. Although the combination of radiotherapy and immunotherapy has demonstrated clinical promise in treating LARC, the influence of treatment sequencing on efficacy appears modulated by multiple variables, including individual patient characteristics, treatment tolerability, adverse effects, and immunotherapy completion rates. Variability in reported outcomes across studies may reflect heterogeneous interactions among these factors during treatment, thereby highlighting the need for large-scale clinical trials to further elucidate the optimal sequencing strategy.

### 5.2 Patient selection criteria

Differences in patient selection criteria can significantly impact study outcomes. For example, the BFH-NCRTPD trial included only specific subtypes, such as ultra-low rectal cancer, which may limit the generalizability of its results to all patients with LARC. Moreover, the lack of subgroup analyses distinguishing pMMR/MSS from dMMR/MSI-H populations in studies like PANDORA, R-IMMUNE, POLAR STAR, and UNION may have led to an overestimation of overall response rates due to the inclusion of dMMR/MSI-H patients. Consequently, the clinical relevance of these findings for pMMR/MSS-type LARC is substantially limited.

## 6 Conclusion

In the neoadjuvant treatment of LARC, the combination of nCRT and immunotherapy has shown considerable clinical potential by effectively addressing the limitations of conventional nCRT, such as low cCR/pCR rates and poor immunotherapeutic response in pMMR/MSS RC. An increasing body of evidence has suggested that integrating radiotherapy with immunotherapy significantly enhances tumor regression and CR rates while maintaining acceptable safety and tolerability profiles, thereby offering expanded opportunities for implementing the W&W strategy in patients with low RC. Future large-scale clinical trials will be required to validate this novel therapeutic approach’s efficacy and further refine methods for assessing treatment outcomes. Moreover, integrating biomarker-based screening to identify patients most likely to benefit will be essential in developing more effective combination regimens. These promising short-term outcomes provide a basis for further investigation into their potential impact on long-term survival and quality of life in LARC patients.
